# Are Antibiotics Appropriately Dosed in Critically Ill Patients with Augmented Renal Clearance? A Narrative Review

**DOI:** 10.1155/2022/1867674

**Published:** 2022-01-31

**Authors:** Mohammad Sistanizad, Rezvan Hassanpour, Elham Pourheidar

**Affiliations:** ^1^Department of Clinical Pharmacy, School of Pharmacy, Shahid Beheshti University of Medical Sciences, Tehran, Iran; ^2^Prevention of Cardiovascular Disease Research Center, Shahid Beheshti University of Medical Sciences, Tehran, Iran

## Abstract

**Aims:**

Augmented renal clearance (ARC), which is commonly defined as increased renal clearance above 130 ml/min/1.73 m^2^, is a common phenomenon among critically ill patients. The increased elimination rate of drugs through the kidneys in patients with ARC can increase the risk of treatment failure due to the exposure to subtherapeutic serum concentrations of medications and affect the optimal management of infections, length of hospital stay, and outcomes. The main goal of this review article is to summarize the recommendations for appropriate dosing of antibiotics in patients with ARC.

**Methods:**

This article is a narrative review of the articles that evaluated different dosing regimens of antibiotics in patients with ARC. The keywords “Augmented Renal Clearance,” “Critically ill patients,” “Drug dosing,” “Serum concentration,” “Beta-lactams,” “Meropenem,” “Imipenem,” “Glycopeptide,” “Vancomycin,” “Teicoplanin,” “Linezolid,” “Colistin,” “Aminoglycosides,” “Amikacin,” “Gentamycin,” “Fluoroquinolones,” “Ciprofloxacin,” and “Levofloxacin” were searched in Scopus, Medline, PubMed, and Google Scholar databases, and pediatric, nonhuman, and non-English studies were excluded.

**Results:**

PK properties of antibiotics including lipophilicity or hydrophilicity, protein binding, the volume of distribution, and elimination rate that affect drug concentration should be considered along with PD parameters for drug dosing in critically ill patients with ARC.

**Conclusion:**

This review recommends a dosing protocol for some antibiotics to help the appropriate dosing of antibiotics in ARC and decrease the risk of subtherapeutic exposure that may be observed while receiving conventional dosing regimens in critically ill patients with ARC.

## 1. What Is Known? 

Augmented renal clearance (ARC) is a common phenomenon in critical care settings. The incidence of ARC was reported between 14 and 85% depending on the study population and the cutoff value of creatinine clearance (CrCl). The CrCl ≥130 ml/min/1.73 m^2^ has been considered the ARC phenomenon in most studies, although different values have been suggested as well. The elimination rate of drugs, especially hydrophilic antibiotics that are mainly eliminated through the kidney, increased in ARC. According to the effect of ARC on the optimal management of infections, length of hospital stay, and clinical outcomes, determination of the ARC phenomenon is necessary for adjusting the optimal treatment to reduce the risk of subtherapeutic exposure that may be observed while receiving conventional dosing regimens in critically ill patients with ARC.

### 1.1. What Is New?

The higher rate of renal elimination of the medications in ARC impacts the dosing regimens of antibiotics to achieve target pharmacokinetic/pharmacodynamic (PK/PD) indices. There are some review articles aimed to recommend dosing regimens for antibiotics in patients with ARC while reviewing the related articles, but each contains only several antibiotics, particularly B-lactams and vancomycin. We tried to prepare an almost complete article that involved most antibiotics with renal excretion.

## 2. Introduction

Infections are one of the most common problems encountered in critically ill patients and may prolong the hospital length of stay, as well as increasing patient mortality rates [1]. The choice of appropriate antibiotics, early administration, and using appropriate dosing regimens are necessary for optimal management of infections [2, 3], whereas augmented renal clearance (ARC) can increase the risk of treatment failure due to subtherapeutic exposure of antibiotics [1, 4–15]. ARC is usually defined as increased renal clearance above 130 ml/min/1.73 m^2^ [4, 6, 15, 16]. According to the increase of the elimination rate of drugs in ARC, especially hydrophilic antibiotics that are mainly eliminated through the kidney, such as beta-lactams, vancomycin, and aminoglycosides, the optimal management of infections and subsequently the length of hospital stay and outcomes are affected [1, 4–15]. Dosing optimization may be particularly important in infections caused by less-susceptible pathogens, where higher antibiotic exposures may be required for optimal efficacy [10, 17]. Therapeutic drug monitoring (TDM) is a highly recommended method for dosing optimization and individualizing the regimen [3, 5, 17–20]. The major limitation of TDM is the lack of availability in every hospital and for every drug.

To the best of our knowledge, there is no published guideline for drug dosing in patients with ARC. The main goal of this narrative review article is to summarize the recommendations for appropriate dosing of antibiotics in ARC to reduce the risk of subtherapeutic exposure that may be observed while receiving conventional dosing regimens in critically ill patients with ARC.

## 3. Methods

This article is a narrative review of the articles that evaluated different dosing regimens of antibiotics in patients with ARC. The keywords “Augmented Renal Clearance,” “Critically ill patients,” “Drug dosing,” “Serum concentration,” “Beta-lactams,” “Meropenem,” “Imipenem,” “Glycopeptide,” “Vancomycin,” “Teicoplanin,” “Linezolid,” “Colistin,” “Aminoglycosides,” “Amikacin,” “Gentamycin,” “Fluoroquinolones,” “Ciprofloxacin,” and “Levofloxacin” were searched in Scopus, Medline, PubMed, and Google Scholar databases, and pediatric, nonhuman, and non-English studies were excluded.

### 3.1. Quality of the Studies Included

All studies except systematic reviews and meta-analyses and case reports were independently rated for quality by two reviewers using the National Institutes of Health (NIH) Quality Assessment Tools [21]. The studies were assessed with questions appropriate to the study design. We graded the quality of the study as good (G) if its rating was at least 70%, fair (F) if its rating was at least 50%, and poor (P) if its rating was less than 50% (Table 1).

## 4. Results

Studies that described the impact of ARC on the dosing regimens of antibiotics, based on achieving target pharmacokinetic/pharmacodynamic (PK/PD) indices, have increased in recent years. There are some review articles aimed to recommend dosing regimens for antibiotics in patients with ARC, but each contains only a few antibiotics, particularly B-lactams and vancomycin [4, 8, 15, 57, 58]. We tried to prepare an almost complete article summarizing the dosing recommendations for most antibiotics with renal excretion.

### 4.1. Beta-Lactams (B-Lactams)

The pattern of B-lactams activity is time-dependent. The time that the free drug plasma concentration remains above the minimum inhibitory concentration (MIC) of the pathogen (*f*T > MIC) has an essential role in efficacy. In practice, depending on the pathogen and the type of B-lactam, *f*T > MIC for 40–70% of the dosing interval is considered an acceptable PD target [4, 11]. In critically ill patients, *f*T > 4 × MIC has been suggested to improve clinical outcomes [4, 8, 13] (Table 2).

B-lactams are mainly eliminated through the kidneys, and renal function alteration could influence the elimination rate constant and consequently PK/PD parameters of these drugs [4]. Evaluation of the correlation between B-lactam concentration and creatinine clearance (CrCl) showed 55% and 36% subtherapeutic B-lactam levels in ARC patients with *Pseudomonas aeruginosa* and *Enterobacter* spp. infections, respectively [17]. Udy A.A. et al. also reported 82% and 72% of trough levels were less than the MIC and 4 × MIC, respectively, in critically ill patients with ARC treated with empirical doses of B-lactams [29].

Although Udy A.A. et al. reported no statistically significant differences in the outcome of the patients with ARC who received continuous B-lactam infusion compared with those who received intermittent infusion [59], some articles recommended increasing frequency of infusion as well as increasing the dosage to increase achieving the optimal *f*T > MIC in patients with ARC [13, 15]. Continuous infusion (over 24 hr) of conventional doses can improve the optimal exposure to B-lactams in patients with ARC, compared to intermittent (over 30 min) or extended infusion (over 3–4 hr) [5]. Extended infusion of standard doses of B-lactams in patients with ARC resulted in 80% less than 100%*f*T > MIC and 37% less than 50%*f*T > MIC [1]. Reviewing the articles that compared prolonged infusion and intermittent infusion to achieve effective B-lactam exposure and maximal bacterial killing showed that even with prolonged infusion, effective exposure may not be achieved in critically ill patients with ARC [60]. Carrie et al. reported CrCl ≥ 170 ml/min as a sensitive threshold (93%) to predict subexposure to B-lactams despite the continuous infusion of high doses [6]. Hobbs A.L.V. et al. had recommended a dosing nomogram for different antibiotics, including B-lactams, in critically ill patients with ARC according to their PK/PD targets and breakpoints that are included in Table 1 [8].

The impact of ARC on clinical outcomes is evaluated by some studies [6, 7, 23, 24, 28, 59] that some of them denied this association [7, 59] and some of them reported a decrease in clinical response following the increase in CrCl [23, 24, 28, 61, 62].

#### 4.1.1. Meropenem

Higher doses of meropenem have been recommended in ARC patients, and studies showed that conventional regimens of meropenem are suboptimal for patients with ARC [10, 12, 30]. It has been shown that, in septic patients with ARC, 8–12 g meropenem daily is needed to obtain the PD target [12, 30]. In patients with CrCl 60 to 90 ml/min/1.73 m^2^, 6 g meropenem daily has been recommended to achieve the PD target, whereas in patients with CrCl ≥ 90 ml/min/1.73 m^2^, in addition to increased dosage, increasing frequency or prolonging duration of infusion also has been reported [10]. In addition to doses and strategy of infusion, Agyeman A. A. et al. recommended combination therapy with meropenem and ciprofloxacin against *P. aeruginosa* isolates to increase synergistic killing and decrease resistance in patients with ARC [22].

#### 4.1.2. Piperacillin-Tazobactam (PTZ)

PK models of nine dosing regimens of PTZ (intermittent infusion, extended infusion (EI), and continuous infusion (CI) of 16 g, 12 g, and 8 g daily piperacillin) have been assessed as achieving the PD targets in septic patients with different CrCls and for different MICs. The MIC of 16 mg/L has been proposed as the clinical cutoff point of piperacillin for *P. aeruginosa* and the probability of target attainment (PTA) ≥90% for optimal piperacillin regimens. The results showed the PTA was ≥90% for 50% *f*T > MIC in all three CI regimens and EI of 12 and 16 g piperacillin daily, whereas the PTA for 100% *f*T > MIC was ≥90% only in all three CI regimens [9]. Treating the patients with 16 + 2 g/day and 20 + 2.5 g/day PTZ was also associated with 100% *f*T > MIC in 93% and 98% of patients with 130 ≤ CrCl < 200 ml/min and 80% and 90% of patients with CrCl ≥ 200 ml/min, respectively. The daily dose of 20 + 2.5 g PTZ was recommended for patients with CrCl ≥ 170 ml/min to reach the PD target with the highest probability [26]. No intoxication or supratherapeutic levels were reported by 20 + 2.5 g PTZ daily in critically ill patients with ARC [25, 26]. In another PK model study, it is reported that even continuous infusion of higher doses of piperacillin (24 g daily) was insufficient to achieve the acceptable PD target in ARC patients with CrCl ≥ 90 ml/min [27].

### 4.2. Glycopeptides

The antibacterial activity pattern of glycopeptides is both time- and concentration-dependent, and the area under the plasma concentration-time curve (AUC) for 24 hr, relative to the pathogen MIC (AUC0–24/MIC) ratio, is considered the best parameter to predict their antibacterial activity [8, 13] (Table 2). Glycopeptides are hydrophilic agents and are eliminated primarily through the kidney [63], so the change in CrCl can affect the PK/PD parameters of these drugs.

#### 4.2.1. Vancomycin

For vancomycin, the target of AUC0–24/MIC ≥  400 has been proposed to have optimal clinical outcomes [8, 35]. Studies showed the correlation of ARC with the augmented clearance of vancomycin, subtherapeutic serum concentration, and a higher risk of treatment failure [14, 64]. It has been observed that using conventional doses of vancomycin (1000 mg every 12 hr) resulted in 62.9% trough concentrations <10 mg/L. The trough level remained less than 10 mg/L despite increasing the dosage of vancomycin to 1000 mg every 8 hr or 1500 mg every 12 hr [33]. A mean daily dose of 44 ± 9 mg/kg/day vancomycin in trauma patients with ARC was also associated with 54.2% therapeutic trough concentration <10 mg/L [32]. According to TDM data, there was a need for higher doses of vancomycin in patients with ARC compared with non-ARC patients (35.7 mg/kg/day vs. 27.1 mg/kg/day) [34]. A loading dose (LD) of 25–30 mg/kg vancomycin followed by a maintenance dose (MD) of 15–20 mg/kg every 8–12 hr or 45 mg/kg/day for vancomycin is recommended in critically ill patients with ARC [8, 15]. Also, it has been proposed that an LD of 35 mg/kg vancomycin followed by continuous infusion of 35 mg/kg/day needs to keep the vancomycin trough concentration within the target therapeutic range in the CrCl equal to 100 ml/min/1.73 m^*2*^ and higher CrCls need the larger MD to maintain the therapeutic exposure [35]. Fransson et al. reported a case with intracranial infection caused by *Streptococcus intermedius* stating that they did not achieve a stable vancomycin target level until they increased the dose to 1.5 g, four times a day [31]. Baptista et al. published a vancomycin dosing nomogram in septic patients with different CrCls to achieve an ideal trough level of vancomycin on the first day of treatment with 84% success [36].

#### 4.2.2. Teicoplanin

Teicoplanin has approximately 90–95% affinity to binding serum protein, and hypoalbuminemia may cause a low total trough level, whereas the unbound trough concentration is in the therapeutic range (1.5–4.5 mg/L). So in settings of hypoalbuminemia, measuring total and unbound trough levels of teicoplanin is recommended [11, 40, 65]. Although a total trough level ≥15 mg/L is considered an optimal therapeutic concentration of teicoplanin for most infections, higher trough levels are suggested for deep and severe infections [40, 66, 67]. But it should be noted that the levels were kept <60 mg/L to avoid toxicity [40, 42].

The negative association between the trough concentrations of teicoplanin and renal function has been reported [42]. Serum albumin and body weight are also other confounding factors to appropriate dosing of teicoplanin [40]. Evaluation of the impact of LD on the trough concentration of teicoplanin during 10-day treatment showed 25%, 38.9%, and 68.6% of patients who received no LD, low LD (<9 mg/kg), and high LD (≥9 mg/kg) achieved the total trough concentrations ≥20 mg/L, respectively [39]. The PTA on the 3^rd^ and 15^th^ days of treatment with 400, 600, 800, and 1000 mg teicoplanin every 12 hr for four consequent doses followed by the same doses every 24 hr for 12 days was evaluated in different CrCls. The results showed the need for 800 or 1000 mg teicoplanin as the LD to provide a trough concentration ≥15 mg/L on day 3 with a PTA ≥90%. But these doses may cause trough concentrations > 60 mg/L on day 15 even in CrCls >90 mL/min/1.73 m^*2*^ [41]. The administration of 12 mg/kg teicoplanin every 12 hr for five consequent doses and then 12 mg/kg every 24 hr resulted in trough concentrations ≥10 mg/L in 62% of critically ill patients with CrCl > 50 mL/min/1.73 m^*2*^. The clinical effectiveness rate was reported as 88% in these patients [43]. 12 mg/kg teicoplanin for both LD and MD resulted in median total and unbound trough concentrations of 15.9 and 3.7 mcg/ml, respectively, in critically ill patients with pneumonia [45].

Due to the limitations of clinical trials in the field of adequate dosing regimen for teicoplanin in patients with ARC, we also mentioned some studies that have suggestions for teicoplanin dosing in critically ill patients whose risk of ARC is high among them. The recommendations of these articles are summarized in Table 1. Although according to the effect of both ARC and hypoalbuminemia on teicoplanin dosing and high prevalence of the hypoalbuminemia as ARC in critically ill patients, these dosing recommendations may not be an exact guide for teicoplanin dosing in patients with ARC but can be helpful.

No association was reported between teicoplanin-induced nephrotoxicity and its trough concentrations [42]. Teicoplanin trough concentrations ≥20 mg/L compared with <20 mg/L showed no significant difference in the rate of adverse events in patients with MRSA infections [38]. Although no teicoplanin-induced renal impairment was reported in trough concentrations ≥10 mg/L, the hepatotoxicity rate was 10% [43].

### 4.3. Linezolid

The antimicrobial activity of linezolid is time- and concentration-dependent. Besides AUC0–24/MIC and %*f*T > MIC that are correlated with the clinical efficacy of linezolid [46, 68], its trough concentration is also associated with clinical response and adverse events and suggested to be kept in the range of 2–10 mg/L to decrease adverse event incidence [47] (Table 2). Due to the amphiphilicity of linezolid and the limited impact of renal clearance on its excretion (approximately 30%), it does not seem ARC can cause subtherapeutic exposure to linezolid [46, 49, 50, 63]. But some studies reported the significant impact of ARC on PK properties of linezolid [46, 49]. The use of LD or continuous infusion of linezolid was recommended to improve the PTA and efficacy of linezolid in patients with ARC and severe sepsis [49]. The administration of conventional dosing of linezolid results in no PD target achievement in patients with ARC, whereas the PTA was 70% in patients with ARC who received a continuous infusion of 600 mg linezolid every 12 hr. According to Monte Carlo simulation, continuous infusion of a higher dose of linezolid (600 mg every 8 hr) could result in a PTA of 93% in patients with ARC with no linezolid-induced adverse effect [46]. The PTA of different regimens of linezolid was evaluated for the MIC of 0.5 to 4 mg/L. An optimal dosing regimen with adequate exposure was considered the one that its PTA was >90%. Although the continuous infusion of 1200 or 1800 mg/day linezolid was recommended in patients with ARC, it was the optimal dosing for MIC ≤ 0.5 mg/L, 2400 mg/day linezolid was optimal for MIC ≤ 1 mg/L, and none was optimal for MIC ≥ 2 mg/L, whether as an intermittent infusion or a continuous infusion. The trough concentrations >10 mg/L were detected by none of the dosing regimens [47]. The PTA values of 600 mg every 12 hr, 800 mg every 12 hr, and 900 mg every 12 hr of linezolid for the MIC of 2 mg/L in critically ill septic patients were 0.26%, 85.59%, and 98.81%, respectively. The dose of 800 mg linezolid every 12 hr was associated with 33.19% probability of thrombocytopenia, whereas this rate was 51.37% for 900 mg every 12 hr. According to the efficacy and safety data, 800 mg linezolid every 12 hr was recommended in septic patients [48].

### 4.4. Colistin

Colistin is a cationic, lipoprotein, hydrophilic antibiotic [51]. The antibacterial activity of colistin is more concentration-dependent than time-dependent, and the AUC/MIC ratio is considered the best PD index to predict its antibacterial efficacy [15, 63, 69, 70] (Table 2). Colistimitate sodium (CMS), the prodrug of colistin, is primarily eliminated through the kidney, and the increased elimination rate of CMS in ARC could alter the PK properties of colistin due to the reduced systemic bioavailability [51, 63, 70]. Studies showed the need for a longer duration therapy and consequently higher cumulative doses of colistin and higher doses of colistin than conventional regimens in patients with ARC in comparison with other patients [51, 53]. There were two cases (12.5%) with mild to moderate colistin-induced AKI, according to the Acute Kidney Injury Network (AKIN) classification, among patients with ARC who were treated with a longer duration of colistin [51]. The colistin-induced AKI was reported to be 44.3%, according to AKIN criteria, among ARC patients who receive higher doses of colistin, whereas none of them need renal replacement therapy or discontinuing the colistin therapy [53]. A dosing nomogram for colistin was published to achieve the appropriate antibacterial efficacy balanced with the risk of nephrotoxicity in different CrCls. But in CrCl >80 ml/min/1.73 *m*^2^  even by the maximum daily recommended dose of colistin in the nomogram (360 mg or approximately 11 mIU daily), the PTA was <40%. So, combination therapy with colistin in addition to high-dose regimens was recommended for these patients [50, 52].

### 4.5. Aminoglycosides (AGs)

AGs are hydrophilic antimicrobial agents with concentration-dependent killing characteristics. Although Cmax/MIC is considered the best PD index to reflect their bactericidal effect [13, 50, 63], the monitoring of trough concentrations of AGs is also recommended to avoid drug toxicity when the plan of treatment is more than 3–5 days [50] (Table 2). AGs are eliminated predominantly through the kidneys, and their doses need to adjust following the renal function alteration [63, 71]. Some studies evaluated ARC as an effective factor in changing the PK characteristics of AGs [72, 73], but due to the limitations of articles in the field of adequate dosing regimen for AGs in patients with ARC, we also mentioned some studies that have suggestions for AG dosing in critically ill patients whose risk of ARC is high among them.

Carrie et al. used the Monte Carlo simulation to report different dosing regimens of amikacin that are needed to achieve PD targets in different CrCls and for different MICs. An optimal regimen was considered the one that had fractional target attainment (FTA) >85%. They recommended 30–35 mg/kg/day amikacin in patients with ARC especially in the exposure to less-susceptible pathogens. The FTA of 30 mg/kg/day amikacin was 30–40%, 20–30%, and 29% for Cmax/MIC ≥ 8, AUC0–24/MIC ≥75, and trough concentration ≥ 2.5 mg/L, respectively, for an MIC of 8 mg/L. These rates were near 100%, 30%, and 37%, respectively, for 35 mg/kg/day amikacin [54]. The recommended dose for gentamycin and tobramycin was 7 mg/kg/day to achieve the PD targets in patients with ARC [8].

In critically ill patients, 30 mg/kg/day amikacin and 7–10 mg/kg/day gentamycin and tobramycin were recommended as initial empirical doses to achieve the optimal PD targets in severe infections [20, 50].

%*f*T > MIC is another PD index that should be noted to decrease the risk of developing resistant pathogens against AGs. It was shown that *f*T > MIC for less than 60% of AG dosing intervals is associated with resistance development. Thereafter, 12.5 mg/kg amikacin every 12 hr instead of a high-dose extended-interval dosage regimen was recommended to achieve the PD target in the empiric treatment of septic patients with minimizing resistance development. The safety of the two regimens was also compared using neutrophil gelatinase-associated lipocalin (NGAL) during 7 days of treatment, and any significant difference was not detected [55].

### 4.6. Fluoroquinolones (FQs)

Although the bacterial killing property of FQs is concentration-dependent, they also show some time-dependent effects [50]. The AUC0–24/MIC ratio is considered the best PK/PD parameter to predict their antibacterial efficacy and is recommended to be kept more than 125 for ciprofloxacin and more than 80 for levofloxacin to improve outcomes in critically ill patients with Gram-negative infections [8, 13, 50, 63] (Table 2). Among FQs that belong to lipophilic drugs, levofloxacin is the most hydrophilic FQ, and its PK is more affected by renal function alteration [50, 74]. In the comparison of four dosing regimens of levofloxacin to achieve the optimal PD target for different MICs in different CrCls, it was shown none of them was optimal (PTA ≥ 85%) for MIC ≥ 1 mg/L in CrCl ≥ 130 ml/min. But 1000 mg once daily was more effective than other dosing regimens, even 500 mg every 12 hr. Any of these regimens was associated with levofloxacin-related adverse events [56]. 400 mg every 8 hr or 600 mg every 12 hr and 750–1000 mg once daily or 500 mg every 12 hr were recommended as initial empirical doses for ciprofloxacin and levofloxacin in critically ill patients with ARC to achieve optimal PD targets [8, 15, 20]. According to the correlation between AUC0–24/MIC <100 and the risk of developing resistance to FQs, the use of maximum recommended doses of FQs was suggested in critically ill patients to avoid subtherapeutic exposure [50].

## 5. Conclusion

PK properties of antibiotics including lipophilicity or hydrophilicity, protein binding, the volume of distribution, and elimination rate that affect drug concentration should be considered along with PD parameters for drug dosing in critically ill patients with ARC. This review recommends a dosing protocol for some antibiotics to help the appropriate dosing of antibiotics in ARC and decrease the risk of subtherapeutic exposure that may be observed while receiving conventional dosing regimens in critically ill patients with ARC (Table 3).

## Figures and Tables

**Table 1:** A summary of recommendations of studies that have been done to compare different dosing regimens for antibiotics in patients with augmented renal clearance.

Author	Year	Type of the study	Population	Number of patients	CrCl measurement method	ARC definition	Main result	Evaluated and/or recommended regimens	Quality grading
*Beta-lactams*									
Agyeman [22]	2021	Randomized clinical trial	In vitro study	*6 P. aeruginosa* isolates		> 130 ml/min/1.73 m^2^	72-hour static concentration-time-kill study	Meropenem 2 g q8hr as intermittent or continuous infusion plus ciprofloxacin 400 mg q8hr	P
Carrie [23]	2019	Retrospective study	Critically ill patients with HAP/VAP	177	CrCl_24h_	≥ 150 ml/min/1.73 *m*^2^	Efficacy and safety	Recommended: *PTZ*: 20 + 2.5 g daily CI after 4 + 0.5 g LD *Meropenem*: 6 g daily CI or 2 g q8hr EI over 4 hr *Cefepime*: 6 g daily CI after 2 g LD over 30 min *Ceftazidime*: 6 g daily CI after 2 g LD over 30 min	G
Gerlach [24]	2019	Retrospective study	Hospitalized patients with bacteremia or/and pneumonia due to P. aeruginosa	102	CrCl_CG_	> 130 ml/min	*f*T > MIC (>60% for cefepime and >50% for PTZ) (MIC = 8 mg/L for cefepime and 16 mg/L for PTZ) clinical cure	*PTZ*: 4.5 g q8hr as the EI over 4 hr *Cefepime*: 2 g q8hr as the EI over 4 hr Both groups received an LD over 30 min	F
Besnard [25]	2019	Prospective study	Critically ill patients	35 (36 serum concentrations)	CrCl_24h_	≥ 150 ml/min	Piperacillin unbound concentration < MIC (= 16 mg/L for *P. aeruginosa*) toxic cutoff of PTZ (≥150 mg/L)	*PTZ*: 20 + 2.5 g/day over 10 hr infusion (160 mg/ml) after 4 + 0.5 g LD over 60 min	P
Jacobs [17]	2018	Retrospective study	Critically ill patients who received TDM	215 (512 drug concentrations)	CrCl_24h_	≥ 120 ml/min	*f*T > 4×MIC (70% for FEP/CAZ, 50% for PIP, and 40% for MEM)	*Meropenem (MEM)* *Cefepime (FEP)* *Ceftazidime (CAZ)* *Piperacillin (PIP)*	F
Carrie [6]	2018	Prospective observational study	Critically ill patients	79 (235 drug concentrations)	CrCl_24h_	≥170 ml/min	Rate of underdosing (<4 × MIC) clinical failure	*PTZ*: 16 + 2 g daily CI as a 12 hr infusion after an LD of 4 + 0.5 g over 60 min *Cefepime*, *ceftazidime*, *cefotaxime*, *meropenem*: 6 g daily CI as an 8 hr infusion after an LD of 2 g over 60 min	F
Burger [10]	2018	Prospective observational study	ICU-admitted patients	101	CrCl_CG_	≥130 ml/min/1.73 *m*^2^	100% *f*T > MIC in 90% of patients	Recommended: *Meropenem*: 6 g daily with increased frequency of administration or duration of infusion	F
Andersen [9]	2018	Prospective study	Septic patients who were treated empirically with PTZ	22	CrCl_CG_	> 130 ml/min/1.73 *m*^2^	100% and 50% fT > MIC (breakpoint MIC for *P. aeruginosa*: 16 mg/L)	IA of 4 g *piperacillin* q6hr, q8hr, and q12hr, over 3 min EI of 4 g *piperacillin* q6hr (over 3 hr), q8hr (over 4 hr), and q12hr (over 6 hr) CI of 20 g, 16 g, and 12 g *piperacillin* daily after a bolus LD of 4 g	F
Carrie [26]	2018	Prospective study	Critically ill patients	59	CrCl_24h_	> 130 ml/min/1.73 *m*^2^	100% *f*T > MIC	*PTZ*: comparison of two dosing regimens (20 + 2.5 g/day vs. 16 + 2 g/day) that are both 12 hr CI	F
Dhaese [27]	2018	Prospective study	Critically ill patients	110 (270 plasma samples)	CrCl_8h_	> 130 ml/min/1.73 m^2^	100% fT > 4×MIC (MIC ≤16 mg/L for susceptible *P. aeruginosa*)	*Piperacillin*: 24 g daily as CI immediately after 4 g LD	F
Mahmoud [15]	2017	Systemic review				≥130 ml/min		Recommended: *Meropenem*: 2 g q8hr *PTZ*: 4 + 0.5 g q6hr as an EI over 4 hr	
Hobbs [8]	2015	Review article	Critically ill patients with ARC		CrCl_8h_	≥ 130 ml/min/1.73 *m*^2^	*f*T > MIC (60% for FEP, 50% for PTZ, and 40% for MEM)	Recommended: *Meropenem (MEM)*: 2 g q8hr as a 3 hr infusion *PTZ*: 4.5 g q6hr as a 4 hr infusion *Cefepime (FEP)*: 2 g q6-8hr as a 3 hr infusion	
Huttner [7]	2015	Observational prospective cohort study	Critically ill patients	100	CrCl_CG_	> 130 ml/min/1.73 m^2^	Clinical response 28 days after inclusion	*Imipenem/cilastatin*: 500 mg q6hr *Meropenem*: 2 g q8hr *PTZ*: 4 + 0.5 g q8hr *Cefepime*: 2 g q12hr	F
Udy [28]	2015	Prospective study	Critically ill patients with sepsis	48	CrCl_6h_	120 to 300 ml/min	Clinical response	*PTZ*: 4.5 g q6hr as an IA over 20 min	F
Carlier [1]	2013	Prospective study	Critically ill patients	61	CrCl_24h_	> 130 ml/min/1.73 *m*^2^	100% and 50% *f*T > MIC	3 hr infusion immediately after an LD over 30 min of the following: *Meropenem*: 1 g q8hr *PTZ*: 4 + 0.5 g q6hr	F
Udy [29]	2012	Observational study	Critically ill patients who received empirical B-lactam therapy	52 trough concentrations collected for TDM	CrCl_8h_	130 ml/min/1.73 *m*^2^	Trough concentrations less than MIC and 4 × MIC	*Ampicillin*, *dicloxacillin*, *penicillin*, *flucloxacillin*, *piperacillin*, *cephalothin*, *cefazolin*, *ceftriaxone*, *ceftazidime*, *cefepime*, *meropenem*, *ertapenem*	F
Taccone [30]	2012	Case report	A patient with septic shock due to XDR P. aeruginosa	1	CrCl_24h_	>200 ml/min	*f*T > 4×MIC ≥40%	*Meropenem*: 12 g daily (3 g q6hr as a 3 hr EI)	
Tröger [12]	2012	Case reports	Septic patients	2	CrCl_CG_ and CrCl_CKD-EPI_	≥120 ml/min	Trough concentration >4×MIC	*Meropenem*: 1 g q8hr	

*Vancomycin*									
Fransson [31]	2021	Case report		1	CrCl_12h_	≥ 130 ml/min/1.73 *m*^2^	Trough concentrations between 10 and 20 mg/L	Despite using doses from 1.5 g q8hr to 2 g q6hr, a stable vancomycin target level was not achieved until 1.5 g q6hr	
Molina [32]	2020	Retrospective study	Traumatic ICU-admitted patients	119	CrCl_CG_	>105 ml/min	Subtherapeutic trough concentration (<10 mg/L)	Mean daily dose: 44 ± 9 mg/kg/day	F
Mahmoud [15]	2017	Systemic review			CrCl_24h_	≥ 130 ml/min/1.73 *m*^2^		LD: 25–30 mg/kg MD: 45 mg/kg/day q8hr	
Chu [33]	2016	Retrospective study	Patients who received empirical vancomycin therapy	148	CrCl_CG_	≥ 130 ml/min	Subtherapeutic trough concentration (<10 mg/L)	1000 mg q12hr	F
Hirai [34]	2016	Retrospective observational study	Patients who were treated with vancomycin	292 (48 patients with ARC)	CrCl_CG_	≥ 130 ml/min/1.73 *m*^2^	Subtherapeutic trough concentrations (≤ 10 mcg/mL)	TDM is used for optimizing the dose of vancomycin	F
Hobbs [8]	2015	Review article	Critically ill patients with ARC		CrCl_8h_	≥ 130 ml/min/1.73 *m*^2^	Trough concentration between 15 and 20 mg/L AUC/MIC >400	LD: 25–30 mg/kg MD: 15–20 mg/kg q8-12hr	
Robert [35]	2011	Retrospective data collection	Critically ill septic patients	206	CrCl_24h_	100 ml/min/1.73 *m*^2^	Trough concentrations between 20 and 30 mg/L	LD: 35 mg/kg over 180 min MD: at least 35 mg/kg/day as CI	F
Baptista [36]	2014	Two-step study (first retrospective and then prospective)	Critically ill patients	104 patients in total (79 and 25 patients, respectively)	CrCl_8h_	≥ 130 ml/min/1.73 *m*^2^	Trough concentrations between 20 and 30 mg/L	Dosing nomogram for different CrCls: LD: 1000 mg for patients with TBW ≤ 70 kg and 1500 mg for TBW >70 kg MD: 3 to 5.8 g/day as CI for a CrCl of 125 to 350 mg/ml	G

*Teicoplanin*									
Li [37]	2020	Retrospective study	Critically ill patients	55	CrCl_CG_		Trough concentration ≥10 mg/L on days 2 and 4 Clinical response Adverse effects (nephrotoxicity and hepatotoxicity)	LD: 400 mg or 800 mg q12hr for three doses MD: 400 mg or 800 mg q24hr, q48hr, or q72hr according to renal adjustment	F
Ueda [38]	2020	Retrospective study	Patients who were treated with teicoplanin	512 (for safety), 76 (for efficacy)	Estimated GFR by a formula that is developed by the Japanese Society of Nephrology		Clinical response Adverse effects (nephrotoxicity and hepatotoxicity) On day 4 and the end of teicoplanin therapy	LD: 12 mg/kg q12hr for four consequent doses and then 12 mg/kg once daily on day 3 MD: 6.7 mg/kg once daily	F
Kim [39]	2019	Retrospective study	Patients who were treated with teicoplanin ≥72 hr	65 (124 serum concentrations)			Trough concentration ≥10 mg/L and ≥20 mg/L within 10 days	No LD, low LD (<9 mg/kg), and high LD (≥ 9 mg/kg) q12hr for three consequent doses The MD is calculated according to TDM and renal adjustment (q24hr, q48hr, or q72hr)	F
Byrne [40]	2018	Prospective study	Patients with haematologic malignancy	30	CrCl_24h_		Trough concentrations: Total ≥ 20 mg/L Unbound ≥ 1.5 mg/L On days 3 and 7	Recommended: 18–25 mg/kg for both LD (five doses with 12 hr interval) and MD (q24hr) for CrCl ≥ 130 ml/min	F
Cazaubon [41]	2017	Retrospective study	Infected patients with Gram-positive cocci	98	CrCl_CG_CrCl_MDRD_		Trough concentration ≥ 15 mg/L AUC0–24/MIC ≥ 900 AUC0–24/MIC ≥ 1800	Monte Carlo simulation	F
Richards et al. [11]	2015	Review article	Critically ill patients			≥130 ml/min		LD: 800 mg teicoplanin twice a day for four consequent doses MD: 400 mg q12hr	
Byrne [42]	2015	Retrospective cohort study	Patients with haematologic malignancy	104	CrCl_CG_		Total trough concentration Treatment outcomes: nephrotoxicity (according to RIFLE criteria)	LD: intravenous bolus injection of 600 mg (800 mg if TBW >80 kg) q12hr for three consequent doses MD: 600 mg (800 mg) once daily Recommended: LD: 12 mg/kg q12hr for 3–5 consequent doses MD: 12 mg/kg daily	F
Nakamura [43]	2015	Prospective study	Critically ill patients	106	CrCl_24h_		Trough concentration between 15 and 30 mg/L on day 3 Clinical response Adverse effects (nephrotoxicity and hepatotoxicity)	LD: 12 mg/kg q12hr for five consequent doses MD: 12 mg/kg according to TDM and renal adjustment	P
Matsumoto [44]	2013	Retrospective study	Critically ill patients	20			Correlation between teicoplanin LD and trough concentration on day 3 Adverse effects (nephrotoxicity and hepatotoxicity)	11–15 mg/kg q12hr for three consequent doses	P
Mimoz [45]	2006	Prospective study	Critically ill patients	13			Trough concentration ≥20 mg/L	LD: 12 mg/kg q12hr for four consecutive doses MD: 12 mg/kg once daily	F

*Linezolid*									
Barrasa [46]	2020	PK modeling study	ICU-admitted patients	43 (136 plasma samples)	CrCl_10h_	≥ 130 ml/min/1.73 *m*^2^	AUC0–24/MIC > 80 fT > MIC > 85% (target MIC = 2 mg/L)	600 mg q12hr as IA (over 30 min) or CI (50 mg/hr) Recommended: 600 mg q8hr as CI (75 mg/hr)	F
Wang [47]	2020	Prospective multicenter observational study	ICU-admitted patients	117	CrCl_CG_	≥ 120 ml/min/1.73 *m*^2^	AUC0–24/MIC >80 Trough concentration <10 mg/L (for MIC 0.5 to 4)	600 mg q12hr	F
Dou [48]	2020	PK modeling study	Critically ill septic patients	52	CrCl_CG_		AUC0–24/MIC of 100 (to provide a bacterial eradication rate of 80% in septic patients) Safety (thrombocytopenia)	Recommended: 800 mg q12hr	F
Morata [49]	2013	Retrospective study	Patients who received linezolid	78	CrCl_MDRD_	≥ 80 ml/min	Trough concentration <2 mg/L	600 mg q12hr	P

*Colistin*									
Fujii [50]	2020	Review article	Critically ill patients					Recommended: combination therapy with following high-dose colistin in patients with CrCl >80 ml/min/1.73 *m*^2^ LD: 9 mIU MD: 360 mg (11 mIU) daily q12hr, 12 hr after the LD	
Aitullina [51]	2019	Retrospective study	ICU-admitted patients with MDR Gram-negative bacterial infection and at least 72 hr colistin therapy	100	CrCl_CKD-EPI_	≥ 108 ml/min/1.73 *m*^2^	Efficacy Nephrotoxicity	LD: 9 mIU LD MD: 3 mIU q8hr	F
Nation [52]	2017	Four-center observational study	Adult critically ill patients	214	CrCl_CG_	≥ 90 ml/min/1.73 *m*^2^	PTA >80% and <30% for average steady-state concentration of colistin ≥2 and ≥4 mg/L, respectively.	An algorithm for colistin dosing in different CrCls: 360 mg (11 mIU) daily for patients with CrCl >90 ml/min/1.73 *m*^2^ q12hr	F
Dalfino [53]	2015	Prospective observational study	Patients with severe sepsis or septic shock who received colistin >72 hr	70	CrCl_CKD-EPI_	> 130 ml/min/1.73 *m*^2^	Nephrotoxicity Steady-state concentration (target = 2.5 mg/L)	Recommended: LD: 9 mIU MD: 9 mIU/day for CrCl 60–130 ml/min/1.73 *m*^2^ and 12 mIU/day for CrCl > 130 ml/min/1.73 *m*^2^ q12hr, 12 hr after an LD	F

*Aminoglycosides*									
Carrie [54]	2020	Retrospective study	Critically ill patients who received amikacin and underwent TDM	70 (179 serum concentrations(	CrCl_CG_	≥ 130 ml/min/1.73 *m*^2^	Cmax/MIC ≥ 8 AUC0–24/MIC ≥75 Trough concentration <2.5 mg/L (toxic cutoff)	Monte Carlo simulations	F
Fujii [50]	2020	Review article	Critically ill patients				Cmax/MIC ≥ 8–10	*Amikacin*: 30 mg/kg/day q24hr *Gentamycin*: 8 mg/kg/day q24hr *Tobramycin*: 10 mg/kg/day q24hr	
Tängdén [20]	2017	Review article	Critically ill patients with severe infections				Amikacin: Cmax/MIC ≥ 8–10, AUC/MIC >70, Cmin <2 mg/L Gentamycin and tobramycin: Cmax/MIC ≥10, AUC/MIC >70, Cmin <0.5 mg/L	Initial empirical dosage: *Amikacin*: 30 mg/kg q24hr *Gentamycin* and *tobramycin*: 7–10 mg/kg q24hr MD: adjusted doses according to TDM	
Hobbs [8]	2015	Review article	Critically ill patients with ARC		CrCl_8h_	>130 ml/min	Cmax/MIC = 8–10	7 mg/kg/day for *gentamycin* and *tobramycin*	
Najmeddi [55]	2014	Randomized clinical trial	Septic patients who received empirical treatment, including amikacin, against Gram-negative bacteria	40			Cmax >40 and fT > MIC >60% Nephrotoxicity	*Amikacin*: 12.5 mg/kg q12hr instead of 25 mg/kg q24hr	G

*Fluoroquinolones*									
Mahmoud [15]	2017	Review article				≥130 ml/min		Recommended: *Levofloxacin*: 750–1000 mg/day	
Tängdén [20]	2017	Review article	Critically ill patients with severe infections				Ciprofloxacin: AUC/MIC ≥125 and Cmax/MIC ≥ 8 Levofloxacin: AUC/MIC ≥80	Recommended: *Levofloxacin*: 750 mg daily or 500 mg q12hr *Ciprofloxacin*: 400 mg q8hr or 600 mg q12hr Given as initial empirical dosage	
Robert [56]	2016	Observational pharmacokinetic study		35	CrCl_CG_		AUC/MIC ≥ 80	Monte Carlo simulations	F
Hobbs [8]	2015	Review article	Critically ill patients with ARC		CrCl_8h_	>130 ml/min	AUC/MIC ≥125	Recommended: *Ciprofloxacin*: 400 mg q8hr *Levofloxacin*: 750 mg daily	

ARC: augmented renal clearance; CrCl: creatinine clearance; CrCl_24h_: measuring urinary creatinine clearance in the 24-hour urinary collection; CrCl_CG_: estimated creatinine clearance using the Cockcroft–Gault equation; CrCl_CKD-EPI_: estimated creatinine clearance using the CKD-EPI equation; % *f*T > MIC: duration of time that the free drug plasma concentration remains above the minimum inhibitory concentration (MIC) of each pathogen; Cmax/MIC ratio: maximum concentration of antibiotic relative to the pathogen MIC; AUC0–24/MIC ratio: area under the plasma concentration-time curve over 24 hr relative to the pathogen MIC; Cmin: minimum concentration of antibiotic; hr: hours; min: minutes; q: every; IA: intermittent administration; EI: extended infusion; CI: continuous infusion; LD: loading dose; MD: maintenance dose; TBW: total body weight; TDM: therapeutic dose monitoring; XDR: extensively drug-resistant; MDR: multidrug-resistant; PTZ: piperacillin-tazobactam; HAP/VAP: hospital-acquired pneumonia/ventilator-associated pneumonia; G: good; F: fair; P: poor.

**Table 2:** A summary of key pharmacodynamic thresholds of antibiotics [4, 20, 49, 57].

Antibiotics	Bacterial killing characteristics^†^	Pharmacodynamic indices^‡^	Pharmacodynamic targets
Beta-lactams	a	% *f*T > MIC^§^ % *f*T > 4 × MIC (in critically ill patients)	*Penicillin*: 50%–60% *Cephalosporin*: 60%–70% *Carbapenem*: 40%–50%
Aminoglycosides	b	Cmax/MIC ratio	(i) Cmax/MIC ≥8–10 (ii) AUC0–24/MIC >70 (iii) Trough concentration: *Amikacin* < 2 mg/L *Gentamycin* and *tobramycin* <0.5 mg/L
Fluoroquinolones	b	AUC0–24/MIC ratio^¥^	*Ciprofloxacin* ≥125 *Levofloxacin* ≥80
Glycopeptides	c	AUC0–24/MIC ratio	*Vancomycin*: (i) AUC0–24/MIC ≥ 400 (ii) Trough concentrations between 10 and 20 mg/L *Teicoplanin*: Trough concentration: (iii) Total between 20 and 60 mg/L (iv) Unbound ≥ 1.5 mg/L
Linezolid	c	AUC0–24/MIC ratio % *f*T > MIC	(i) AUC0–24/MIC = 80–120 (ii) *f*T > MIC >85% (iii) Trough concentration between 2 and 10 mg/L
Colistin	c	AUC0–24/MIC ratio	Steady-state concentration ≥ 2 mg/L

^†^Bacterial killing characteristics of antibiotics: a: time-dependent agents, b: concentration-dependent agents, and c: concentration- and time-dependent agents; ^‡^pharmacodynamic indices: best parameters to predict the antibacterial activity of antibiotics and their correlation with clinical efficacy; ^§^% *f*T > MIC: duration of time that the free drug plasma concentration remains above the minimum inhibitory concentration (MIC) of each pathogen; Cmax/MIC ratio: maximum concentration of antibiotic relative to the pathogen MIC; ^¥^AUC0–24/MIC ratio: area under the plasma concentration-time curve over 24 hr relative to the pathogen MIC.

**Table 3:** Our recommendations for the antibiotic dosing regimen in patients with augmented renal clearance (CrCl ≥ 130 ml/min/1.73m^2^).

Antibiotics		Dosing regimens
Beta-lactams	Meropenem	2 g every 6–8 hr as a prolonged infusion
	Cefepime	
	Ceftazidime	2 g every 8 hr as a prolonged infusion
	Piperacillin-tazobactam	LD: 4 + 0.5 g over 30 min MD: 20 + 2.5 g daily as a prolonged infusion; it seems better to choose other options for treatment of patients with ARC if possible [24, 27]

Glycopeptides	Vancomycin^‡^	LD: 35 mg/kg MD: 15 mg/kg every 8 hr as a prolonged infusion
	Teicoplanin^‡^	LD: 12 mg/kg (800 mg) every 12 hr for five consequent doses MD: 12 mg/kg mg (800 mg) once daily

Linezolid		800 mg every 12 hr as a continuous infusion
Colistin		LD: 9 mIU MD: 12 mIU/day every 12 hr over 1–2 hr infusion

Aminoglycosides	Gentamycin^§^	7–10 mg/kg/day every 12 hr
	Tobramycin^§^	
	Amikacin^§^	30 mg/kg/day every 12 hr

Fluoroquinolones	Ciprofloxacin	400 mg every 8 hr
	Levofloxacin	1000 mg once daily

CrCl: creatinine clearance; LD: loading dose; MD: maintenance dose; ^‡^based on the total body weight (TBW); ^§^based on the adjusted body weight (ABW = ideal body weight (IBW) + 0.4 (TBW–IBW)).

## Data Availability

The data supporting this narrative review are from previously reported studies and datasets, which have been cited. The processed data are available upon request from the corresponding author.
